# Structure of the *Streptococcus pneumoniae* Surface Protein and Adhesin PfbA

**DOI:** 10.1371/journal.pone.0067190

**Published:** 2013-07-22

**Authors:** Michael D. Suits, Alisdair B. Boraston

**Affiliations:** 1 Department of Biochemistry and Microbiology, University of Victoria, Victoria, British Columbia, Canada; University of Canterbury, New Zealand

## Abstract

PfbA (plasmin- and fibronectin-binding protein A) is an extracellular *Streptococcus pneumoniae* cell-wall attached surface protein that binds to fibronectin, plasmin, and plasminogen. Here we present a structural analysis of the surface exposed domains of PfbA using a combined approach of X-ray crystallography and small-angle X-ray scattering (SAXS). The crystal structure of the PfbA core domain, here called PfbAβ, determined to 2.28 Å resolution revealed an elongated 12-stranded parallel β-helix fold, which structure-based comparisons reveal is most similar to proteins with carbohydrate modifying activity. A notable feature of the PfbAβ is an extensive cleft on one face of the protein with electrochemical and spatial features that are analogous to structurally similar carbohydrate-active enzymes utilizing this feature for substrate accommodation. Though this cleft displays a combination of basic amino acid residues and solvent exposed aromatic amino acids that are distinct features for recognition of carbohydrates, no obvious arrangement of amino acid side chains that would constitute catalytic machinery is evident. The pseudo-atomic SAXS model of a larger fragment of PfbA suggests that it has a relatively well-ordered structure with the N-terminal and core domains of PfbA adopting an extend organization and reveals a novel structural class of surface exposed pneumococcal matrix molecule adhesins.

## Introduction

*Streptococcus pneumoniae* (the pneumococcus) is a transient colonizer of the human nasopharynx. Though this is typically a benign relationship, the bacterium can, under appropriate but not entirely understood circumstances, switch into the role of a proficient invasive pathogen. Mediating the host-bacterium interaction is the complex extracellular landscape of the pneumococcus that includes both covalently and non-covalently linked proteins that perform a wide variety of functions [1]. Approximately fifteen of these proteins are known or predicted to be attached to the bacterial cell-wall through a sortase-dependent mechanism requiring an LPXTG sequence motif in addition to a secretion signal peptide [2]. In *S. pneumoniae* TIGR4 these specific cell-surface attached proteins comprise seven carbohydrate-active enzymes, four proteases, one mucin specific adhesin, two adhesins with fibronectin binding ability, and one protein of unidentified function [3]. Additionally, a key role for microbial surface components recognizing adhesive matrix molecules has emerged as a central theme for pneumococcal pathogenesis in that six proteins, PavA, PavB, PepO, PfbA, PfbB (recently reviewed in [4]), and RrgA [5] have all been identified to mediate pneumococcal attachment to plasminogen and fibronectin. Given the important biological functions of these surface exposed proteins in the host-bacterium interaction, and the potential they hold in developing vaccine components their structures and functions hold considerable practical therapeutic interest.

PfbA (plasmin- and fibronectin-binding protein A) is one of the most recently identified surface attached pneumococcal proteins. The gene encoding this protein is highly conserved across all sequenced pneumococcal isolates, and PfbA was shown to be a constitutively expressed, surface-anchored protein [6]. Recombinant PfbA was found to bind to the human extracellular matrix proteins fibronectin and plasmin with high affinities (K_D_ of 4.1 and 2.4 µM, respectively) [6], and may therefore be classified into the microbial surface cell recognition adhesion matrix molecule (MSCRAMM) family [3], [4]. Mutants of *S. pneumoniae* R6 lacking the *pfbA* gene were deficient in the capacity for adherence to, and invasion of, human epithelial cells. Moreover, in keeping with the *in vitro* binding capacity of recombinant PfbA, the adherent and invasive potential of *S. pneumoniae* R6, but not the Δ*pfbA* mutant, was shown to be dependent on the presence of fibronectin. Together, these results indicate that PfbA may be considered an important adhesin in mediating the pneumococcus-host interaction. A full understanding of the biological role of this protein, however, is presently hampered by a lack of structural information. To this end, we have determined the crystal structure of the 422 amino acid core PfbAβ domain (residues 139–560). Greater insight into the overall structure of the protein is provided by a solution SAXS analysis of a 509 amino acid multi-domain construct of PfbA that includes the PfbAβ and an N-terminal domain of unknown function. Together, the results reveal a protein with an extended architecture that contains a core parallel β-helix structure with specific molecular features that we propose are most consistent with the recognition and perhaps processing of carbohydrates.

## Results and Discussion

### PfbA architecture

**Figure 1:**
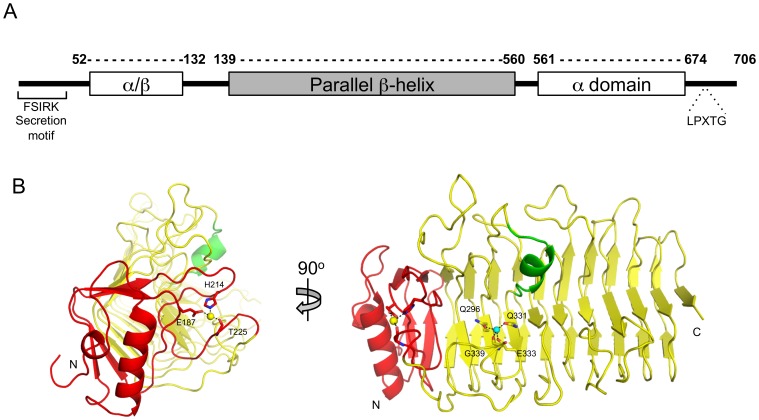
Schematic representation of the modular architecture of PfbA and structure of the parallel β-helix core domain. (A) The PfbA architecture include the FSIRK and LPXTG sequence motifs that are common Gram-positive export signal peptide and streptococcal cell-wall adherence features, respectively. The surface exposed α/β and cell-wall link domains are conserved across pneumococcal isolates. (B) Cartoon representation of the CAZyme-like 12-stranded β-helix domain solved by X-ray crystallography to 2.28 Å. The 90 residue (T139-Q230, coloured red) N-terminal initiator of the core domain is a conserved precursor of the parallel β-helix fold and includes the calcium-binding site established by the sidechains of E187 and H214 and the carbonyl oxygen of T225. A water coordinated by residues Q296, Q331, E333 and the carbonyl oxygen of G339 is reminiscent of the metal binding sites seen in polysaccharide lyases.

A gene encoding PfbA is present amongst the majority of pneumococcal strains with the encoded proteins having no less than 99% amino acid sequence identity. Furthermore, homologues of this protein are distributed across a number of streptococcal and staphylococcal species. The N-terminal FSIRK and C-terminal LPXTG sequence motifs of PfbA are common Gram-positive export signal peptide and sortase-mediated cell-wall attachment motifs, respectively, consistent with the cell-surface localization of the protein [6]. To gain more insight into the architecture of the protein we initially used an approach that combined secondary structure prediction with fold recognition [7], which readily revealed a central domain in PfbA that likely adopts a parallel β-helix fold (residues 139–560) (Figure 1A). This is consistent with distant amino acid sequence similarity between PfbA and various carbohydrate-active enzyme families including polysaccharide lyase families 1, 3, 6 and 9, and glycoside hydrolase families 28 and 49, and 55, all of which adopt parallel β-helix folds [8]. Because of this predicted sequence and fold similarity, PfbA and its homologues have been placed into the “non-classified” hydrolase category within the Carbohydrate-Active Enzyme Database [8]. In addition to the dominant β-helix domain, PfbA contains a predicted N-terminal mixed/β-domain (residues 52–138) and a predicted C-terminal, α-helical bundle (residues 561–708) (Figure 1A). Guided by this predicted domain architecture we generated constructs of the protein by dissecting a synthetic gene optimized for expression in *Escherichia coli*. With this approach we were able to produce sufficient quantities of two proteins comprising only the β-helix domain, PfbAβ (amino acids 139–560), and the β-helix domain with the N-terminal α/β-domain, PfbAΔC (amino acids 52–560), and use these for structural studies.

### X-ray crystal structure of the PfbAβ

We successfully crystallized PfbAβ in two crystal forms. The structure was eventually solved by the single wavelength anomalous dispersion method using selenomethionine labeled protein in the P2_1_ space group with four protein molecules in the asymmetric unit. Strong diffraction anisotropy (typical anisotropic ΔB ranges >30 Å^2^) were characteristic of the data and combined with suboptimal completeness confounded reduction of R-factors during structural refinement in this crystal form, even when anisotropic diffraction corrections were applied. Therefore, diffraction data from the C222_1_ crystal form that contained only one monomer in the asymmetric unit was ultimately used to complete and refine the atomic model of PfbAβ to R_work_ and R_free_ values of 20.9 and 25.3%, respectively (Table 1). An examination of the crystal packing in both crystal forms did not indicate any molecular interfaces of substantial surface area between PfbA monomers that might constitute the formation of biologically relevant quaternary structure.

**Table 1:** X-ray data collection, processing and PfbAβ model refinement statistics.

Data collection statistics	SeMet Peak-Tetramer	Native-Monomer
Wavelength	0.97908	0.97949
Beamline	SSRL 9-2	CMCF-BM
Space group	P2_1_	C222_1_
Resolution	50-2.20 (2.28-2.20)	38-2.25 (2.40-2.28)
Cell dimension	114.4, 62.8, 128.5	62.8, 140.7, 128.6
α, β, γ (Å)	90.0, 106.2, 90.0	90.0, 90.0, 90.0
*R_merge_*	0.113 (0.469)	0.133 (0.503)
Completeness (%)	95.9 (93.8)	100(100)
*<I/σI>*	15.0 (3.9)	6.7 (2.4)
Redundancy	12.8 (10.6)	5.5 (5.5)
Total reflections	165607	462327
Unique reflections	88996	26446
*Refinement statistics*		
*R_work_* (%)		20.8
*R_free_* (%)		25.5
RMSD		0.013
Bond lengths (Å)		1.666
Bond angles (°)		
Number of Atoms		
Protein Chain		3257
Water molecules		59
Average *B*-factors (Å^2^)		
Protein Chain		42.2
Water molecules		42.7
Ramachandran statistics		
Most favored (%)		91.6
Additional allowed (%)		7.2
Disallowed (%)		1.2

PfbAβ displays a right-handed, parallel 12-stranded β-helix fold that is preceded at the N-terminus by a helix-loop-helix arrangement comprising residues 139–171. This motif is followed by a calcium-binding site, which appears to stabilize the initiation of the β-helix and is established by sidechain contributions from E187 and H214, and the main chain carbonyl oxygen of T225. Together these two N-terminal features delineate a 90 residue structural motif (Figure 1B) that is distinctive of carbohydrate-active enzymes possessing β-helix folds. For example, this motif in PfbAβ has high structural similarity with same feature in the *Azotobacter vinelandii* mannuronan C-5 Epimerase AlgE4 (r.m.s.d. 1.19 Å with PDBID: 2PYH over 79 residues) [9] and in the *Alteromonas fortis* iota-carrageenase (r.m.s.d. 1.37 Å with PDBID: 1H80 over 78 residues) [10]. However, beyond providing a general structure-stabilizing role and putative folding scaffold for initiation of the β-helix, the functional significance of this structural motif is unknown.

Further linking the structural relationship of PfbAβ with carbohydrate-active enzymes is significant structural identity with the *endo*-*N*-acetylglucosaminidase tailspike protein from the *E. coli* bacteriophage HK620 (r.m.s.d. = 2.08 Å with PDBID: 2VJJ over 308 residues) [11], the *Pedobacter heparinus* Chondroitinase B (r.m.s.d. = 2.18 Å with PDBID: 1OFL over 278 residues) [12], the Bacillus sp. snu-7 inulin fructotransferase (r.m.s.d. = 2.18 Å with PDBID: 2INV over 245 residues) [13] and the pectate lyase Pel9A from *Erwinia chrysanthemi* (r.m.s.d. = 2.37 Å with PDBID: 1RU4 over 254 residues) [14] (Figure S1). PfbAβ also shows structural identity with a variety of other parallel β-helix proteins, such as the *Bordetella pertussis* virulence factor P.69 pertactin (r.m.s.d = 2.69 Å with PDBID: 1DAB over 255 residues) [15]. However, as the parallel β-helix can vary widely in the number of β-strands used to form the helical structure, and in the loop structures that decorate this general fold, PfbAβ typically only superposed with a fragment of these larger structures and yielded no functional insight. This structural comparison revealed that not only is the general β-helical fold and the number of β-strands employed best conserved with carbohydrate-active enzymes but so are other key features, such as the metal binding cap motif discussed above, which appears to be a distinctive modification of carbohydrate-active enzymes adopting a parallel β-helix fold, and the loops that contour their active sites.

**Figure 2:**
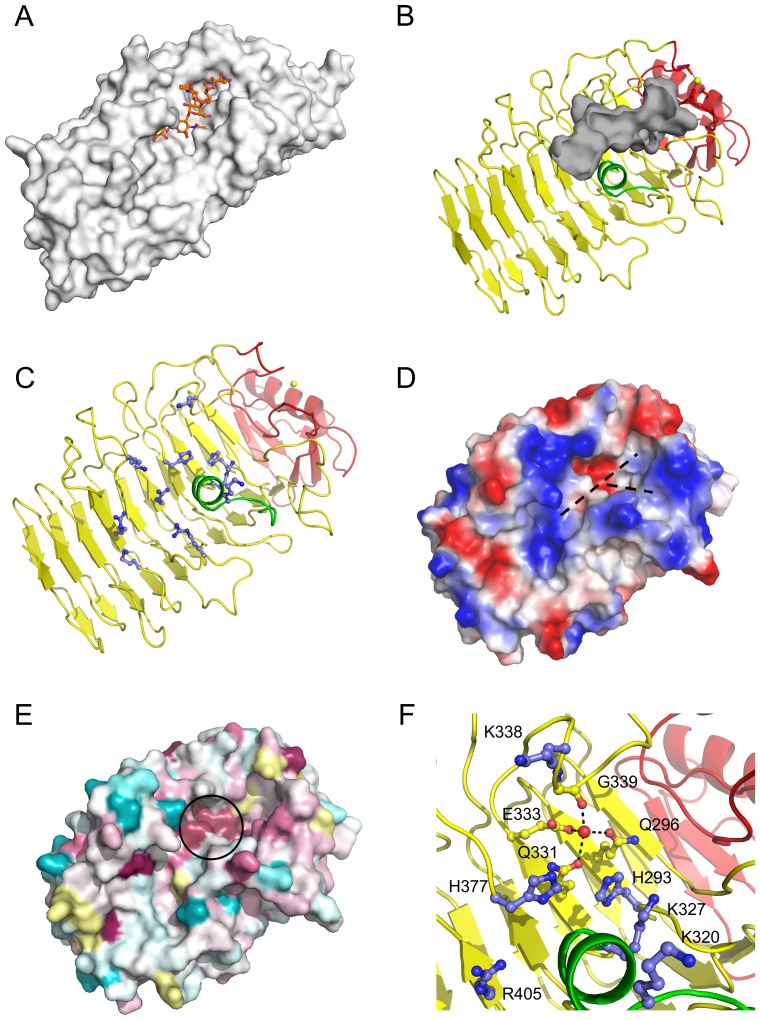
PfbAβ surface features and putative carbohydrate accommodation Cleft. (A) Carbohydrate accommodation groove of *endo*-*N*-acetylglucosaminidase tailspike protein from the *E. coli* bacteriophage HK620 complexed with the substrate *O*-antigen hexasaccharide (orange). (B) A putative carbohydrate accommodation groove (grey) formed along the length of the PfbAβ domain has features conserved across carbohydrate processing factors. (C) A series of electropositive residues (blue) line this cleft (dashed line) which could interact with negatively charged polysaccharides. (D) The solvent accessible surface of PfbAβ colored by electrostatic potential also reveals the electropositive nature of the cleft and shows the branched nature of the cleft. (E) Phylogenetic mapping of homologous sequences to PfbA. Conserved residues are shown in purple and pink, neutral in white, ambiguous in yellow, and non-conserved in blue as per Consurf standard colouration scheme. (F) The electronegative residues Q296, Q331, E333 and carbonyl G339 coordinate a crystallographic water, are conserved at the base of the cleft branch point. This putative active centre is lined with the electropositive series of residues H293, K320, K327, K338, H377 and R405 which have features similar to carbohydate active lyases. This conserved region is circled in panel E.

While the parallel β-helix carbohydrate-active enzymes are quite diverse in their carbohydrate specificity and catalytic mechanisms, they converge structurally in that the groove established along the β-helix serves to accommodate their respective carbohydrate substrate (Figure 2A and Figure S1). PfbAβ possess a distinct groove on its surface that is conserved in location with the substrate binding grooves of β-helix carbohydrate-active enzymes (Figure 2B). The PfbAβ groove runs the length of the protein face, ∼50 Å, is open at one end and ends blindly where it is blocked by the N-terminal helix-turn-helix motif and calcium-binding site (Figure 2B). The PfbAβ groove is most pronounced over ∼28 Å, up to the blind ending (Figure 2B), and over this stretch is lined with a distinct series of electropositive residues (H293, K320, K327, H377, R405, R431, K444, K471, and R474) (Figure 2C) and tyrosine residues (Y378, Y401, Y441 and Y443). The groove is slightly kinked due to the intrusion of an α-helical segment contributed by residues L315-K327 (Figure 2B). At the blind end of the cleft it branches to create two terminal pockets with volumes of ∼110 and 90 Å^3^, respectively (Figure 2D).

These features suggest several additional similarities with carbohydrate-active enzymes. First, the dimensions of the groove are consistent with the accommodation of a polysaccharide or glycan chain, like the CAZymes with which PfbAβ shares structural identity. Second, the electropositive nature of the groove suggests recognition of an acidic molecule, such as glycosaminoglycans, and is indeed reminiscent of the active site grooves of polysaccharide lyases that are active on acidic polysaccharides. Last, there is a sequence-conserved motif comprising Q296, Q331, E333 and G339, which coordinates a well-ordered water (Figure 2E and 2F). Though this site resembles a metal binding site, interatomic distances averaging ∼2.7 Å between the coordinated atom and ligand oxygens, along with a refined B-factor of 47 Å^2^ for a modeled water molecule, are most consistent with this atom being a water. Given the all oxygen nature of the ligands and the frequency of calcium atoms in carbohydrate-active enzyme active sites we attempted several soaks of the crystals in excess calcium chloride but failed to achieve occupation of this site by anything other than a water molecule (not shown). This site is flanked by a proximal series of basic residues: H293, K338, and H377 (Figure 2F), a feature that is again evocative of the catalytic machinery used by polysaccharide lyases employing a β-elimination mechanism of bond cleavage [14], [16], [17]. However, it is clear that the motif does not coordinate the key metal ion common to polysaccharide lyases, rather it is a water, and the spatial arrangement of basic residues does not match that of the catalytic residues in known polysaccharide lyases. Consistent with this when we tested PfbAΔC for polysaccharide lyase activity on a panel of polysaccharides including chondroitin A, B, and C, heparin, hyaluronan, pectic galactan, and polygalacturonic acid, at various pH values between 5.5 and 9, and in the presence and absence of 5 mM CaCl_2_ or MgCl_2_ we did not detect any activity. Furthermore, using isothermal titration calorimetry and affinity gel electrophoresis we could not detect any binding to the glycosaminoglycans. Thus, though PfbAβ displays significant structural similarities to polysaccharide lyases it does not appear to share their function.

The known ligands of PfbA - fibronectin, plasmin, and plasminogen - are quite diverse at the polypeptide and structural levels. Putatively, as they are all glycoproteins, the carbohydrate decorations could serve as a feature recognized by PfbA. Given the apparent lack of activity on glycosaminoglycans, but the inescapable structural similarity of PfbAβ to proteins that recognize carbohydrates, and the shared presence of glycans on the known ligands of PfbA, we presently favour the hypothesis that the parallel β-helix domain of PfbA recognizes a glycan decoration present on glycoconjugates. Unfortunately, repeated screening for binding on glycan microarrays has resulted in inconclusive outcomes.

### Solution conformation of PfbA

PfbA is relatively small at 708 amino acids compared to many of the surface attached proteins in the pneumococcus, such as BgaA, which is over 2200 amino acids, but it nevertheless also displays a multimodular architecture. The PfbAβ core for which we determined the crystal structure represents only ∼60% of the entire protein and larger fragments of PfbA were resistant to crystallization. To provide greater insight into the overall conformation of this surface-presented adhesin we utilized SAXS to analyze the solution conformation of PfbAΔC.

Highly purified PfbAΔC was analyzed by dynamic light scattering (DLS) prior to analyses by solution SAXS. The analysis yielded a consistent radius of 4.37 (±0.06) nm while the baseline and SOS values of 1.0000 (±0.001) and 1.12 (±0.73), respectively, were highly reproducible across the range of protein concentrations and were consistent with a monodisperse protein population [18]. Polydispersity values (%Pd) were also consistent at 7.3 (±1.9) % and typical for a monodisperse protein population [18]. These DLS results interpreted together suggest a non-aggregated monomeric population.

**Figure 3:**
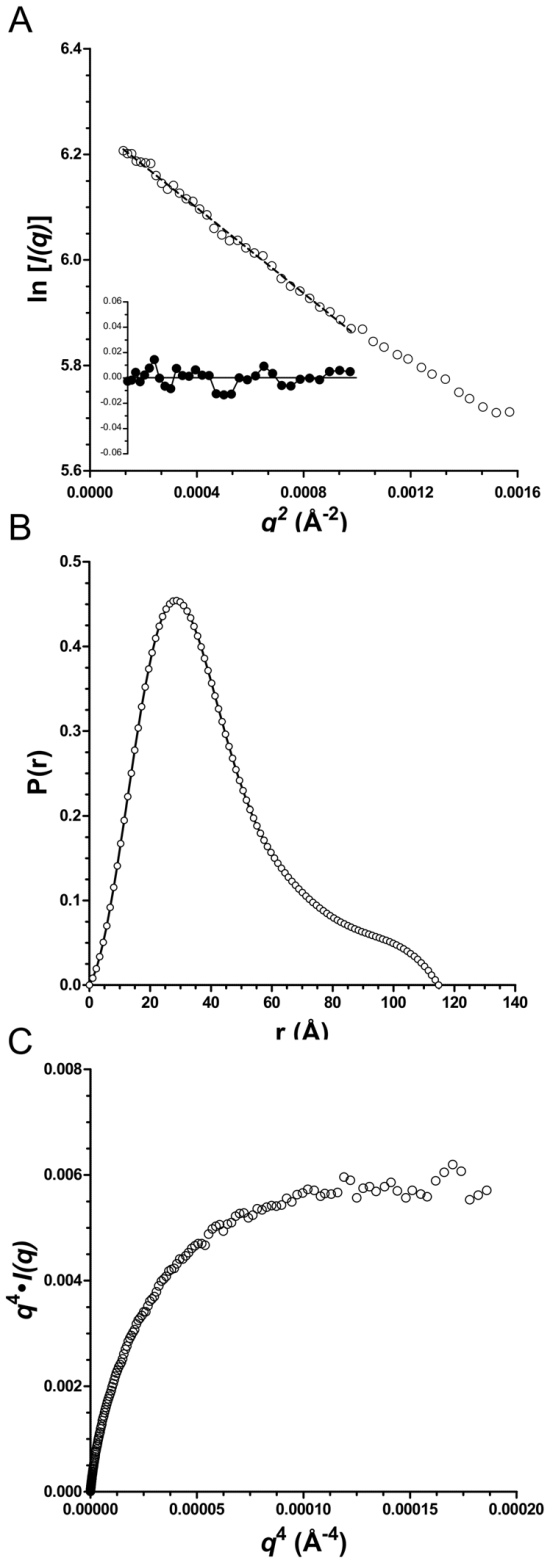
PfbAΔC SAXS data analysis. (A) GUINIER plot and residual of scattering data of PfbAΔC at 1.0 mg/ml are consistent with a radii of gyration of 34 Å. (B) The GNOM generated P(r) distribution of PfbAΔC at 1.0 mg/ml is asymmetric with a short tail extending towards a D_max_ approaching 120 Å. (C) Porod-Debye plots of PfbAΔC SAXS data at 1.0 mg/ml displays the characteristic asymptote of a well structured protein.

Consistent with the DLS, the X-ray scattering profiles obtained for a range of PfbAΔC concentrations (1.0 to 2.5 mg/ml) were characteristic of monodisperse samples [19]. Supporting this is an average particle density calculated from the Porod volumes of 1.18 (±0.03) g/cm^3^, which is consistent with that expected for well-structured proteins [20]. The radii of gyration (R_g_) derived from the Guinier plots (Figure 3A) [21] were, within error, the same at approximately 34 Å indicating the R_g_ is concentration independent over the range tested [22] (Table 2). The analysis of the SAXS data using the program GNOM [23] gives R_g_ values with a similarly narrow range. The pair distance distribution function P(r) computed with GNOM gave an asymmetric distribution with a short tail extending towards a D_max_ approaching 120 Å (Figure 3B and Table 2). A Porod-Debye plot of *q* ^4^⋅*I(q)* vs. *q* ^4^ displays the characteristic asymptote of a well-folded protein (Figure 3C) [20].The experimental molecular weight determined by the method of Fischer *et al.* [24] gave a range of MWs from 65 to 67.7 kDa (Table 2), which are slightly higher than the expected MW of 58.0 kDa but within a tolerance of 15% error.

**Table 2:** SAXS statistics.

							*Ab initio* modelling	
Concentration	Molecular weight kDa^a^	Number of amino acids	R_g_ (Guinier) Å	R_g_ (GNOM) Å	Porod Volume Å^3^	D_max_ Å	χ(DAMMIF)^b^	NSD^c^
**PfbAΔC**	61.2*	565						
2.5 mg.ml^−1^	67.7		35.0±3.2	35.2	86320	120	1.60	0.56±0.01
1.75 mg.ml^−1^	66.0		34.8±2.2	35.1	88430	120	1.56	0.53±0.02
1.0 mg.ml^−1^	65.0		32.8±1.2	33.3	84500	115	1.51	0.56±0.01
**BSA**	66.4*	607						
4.8 mg.ml^−1^	67.2		33.9±0.3	32.4	123700	95	N/A	N/A

* Theoretical molecular weight.

a calculated by the method of Fischer *et al.* [24].

b average of χ-values determined for the 20 models calculated by the DAMMIF *ab initio* modelling procedure [25]. Standard deviations calculated for the χ-values were <0.001.

c averaged normalized spatial discrepancies (NSD) for the 20 models calculated by the DAMMIF *ab initio* modeling procedure [44].

**Figure 4:**
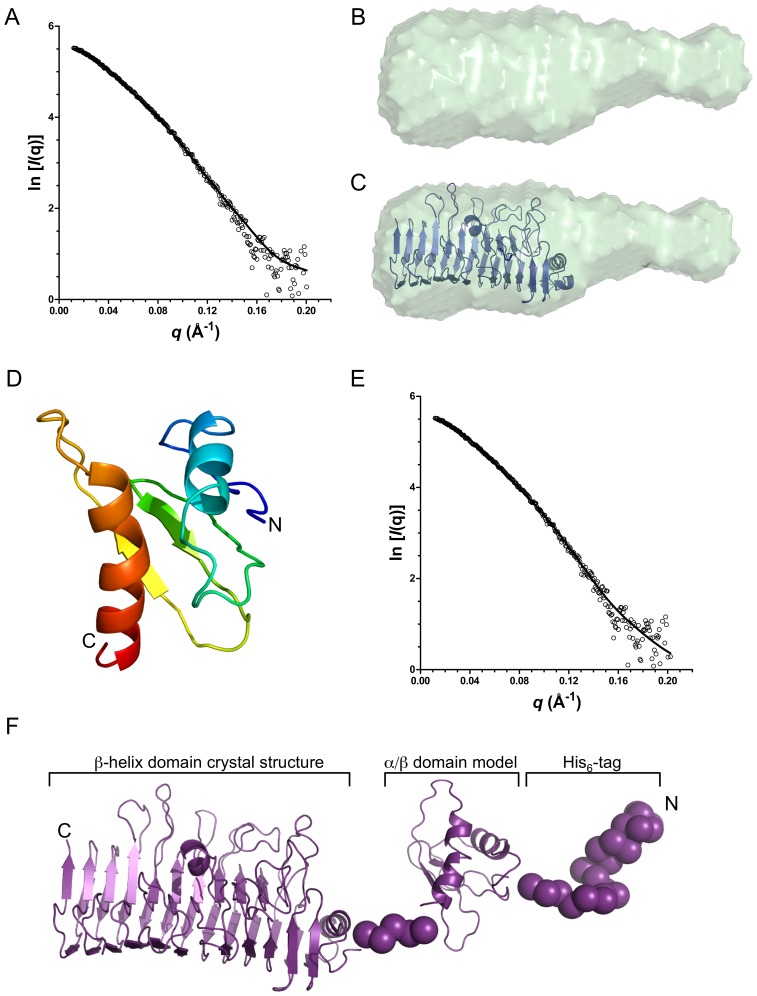
PfbAΔC pseudo-atomic solution model. (A) Experimental and theoretical SAXS data calculated for the (B) averaged *ab initio* surface representation of 10 independent DAMMIF calculations and (C), rigid body fit of the PfbAβ domain into the envelope. (D) I-TASSER generated model of the PfbA N-terminal α/β domain (residues 54–138). (E) CRYSOL calculated and experimental SAXS data for the (F) PfbAΔC composite structure generated from SAXS data with the program BUNCH. Individual models resulted in excellent fits to the data with χ_(DAMMIF)_ values in the range of 1.5–1.6.

*Ab initio* bead models of PfbAΔC were generated from the SAXS data with the program DAMMIF [25]. Twenty models were generated for each concentration of PfbA with the individual models resulting in good fits to the data with χ_(DAMMIF)_ values in the range of 1.5–1.6 (Table 2 and Figure 4A). Averaging of the resulting 20 models for each concentration gave quite small normalized spatial discrepancies, reflecting the consistency between individual models for a given concentration, and an averaged envelope resembling the asymmetric shape of a bottle (Table 2 and Figure 4B). The parallel β-helix of the PfbAβ clearly fits the large lobe of the shape revealing the general position of the remaining N-terminal mixed α/β portion of the protein as it is in solution (Figure 4C).

To add to our understanding of the surface exposed conformation of PfbA we utilized the SAXS data (1.0 mg/ml) for rigid body modeling with the program BUNCH to build pseudo-atomic models of PfbAΔC. As the structure of the N-terminal 84 amino acid domain in the PfbAΔC construct was of unknown structure we used I-TASSER to predict the tertiary structure of this domain; the resulting fold was a pair of α-helices packed against a 2 or 3 stranded β-sheet arrangement (Figure 4D). The SAXS modeling procedure used the X-ray crystal structure of PfbAβ and the predicted model of the N-terminal domain as separate rigid bodies; the 5 amino acid linker between the domains and the 25 amino acid N-terminal sequence that includes the His_6_-purfication tag were modeled as dummy atoms. This modeling analysis was performed over 10 iterations with virtually identical models resulting across the runs. The theoretical scattering of a representative model fit to the experimental data resulted in a χ_crysol_ value of 1.6 (Figure 4E). The overall model reveals an extended conformation with a maximum dimension of ∼127 Å consistent with the shape and dimensions obtained from the *ab initio* modeling of PfbAΔC (Figure 4F).

A highly common feature of *S. pneumoniae* surface proteins is multimodularity [1], which is indeed a very frequent property of carbohydrate-active enzymes in general [26]. In such proteins the modules are commonly structurally and functionally independent allowing a “dissect and build” approach to understanding the individual functions of the composite modules in a protein [26]. PfbA falls into this class of multimodular protein with three modules that can be defined on the basis of bioinformatics analyses (Figure 1). Here we were able to produce large quantities of stable PfbAβ and PfbAΔC and demonstrated through structural approaches that the PfbAβ and PfbAΔC are well-folded polypeptides with structurally distinct modules, consistent with the typical pattern of a multimodular protein with independent modules. Were unable to find a distinct enzymatic or adhesive function for the PfbAΔC construct of PfbA, raising the question of whether the uncharacterized C-terminal α-domain is a necessary for activity of the protein. Given the structural integrity of the PfbAβ and PfbAΔC constructs, the high structural identity of CAZymes with the entire parallel β-helix module (PfbAβ) for which a carbohydrate active function was hypothesized, and the general functional independence of modules in multimodular proteins, we believe it highly unlikely that the observed lack of activity of PfbAΔC in our assays was specifically due to the lack of the C-terminal α-domain. We cannot fully discount this possibility, however.

**Figure 5:**
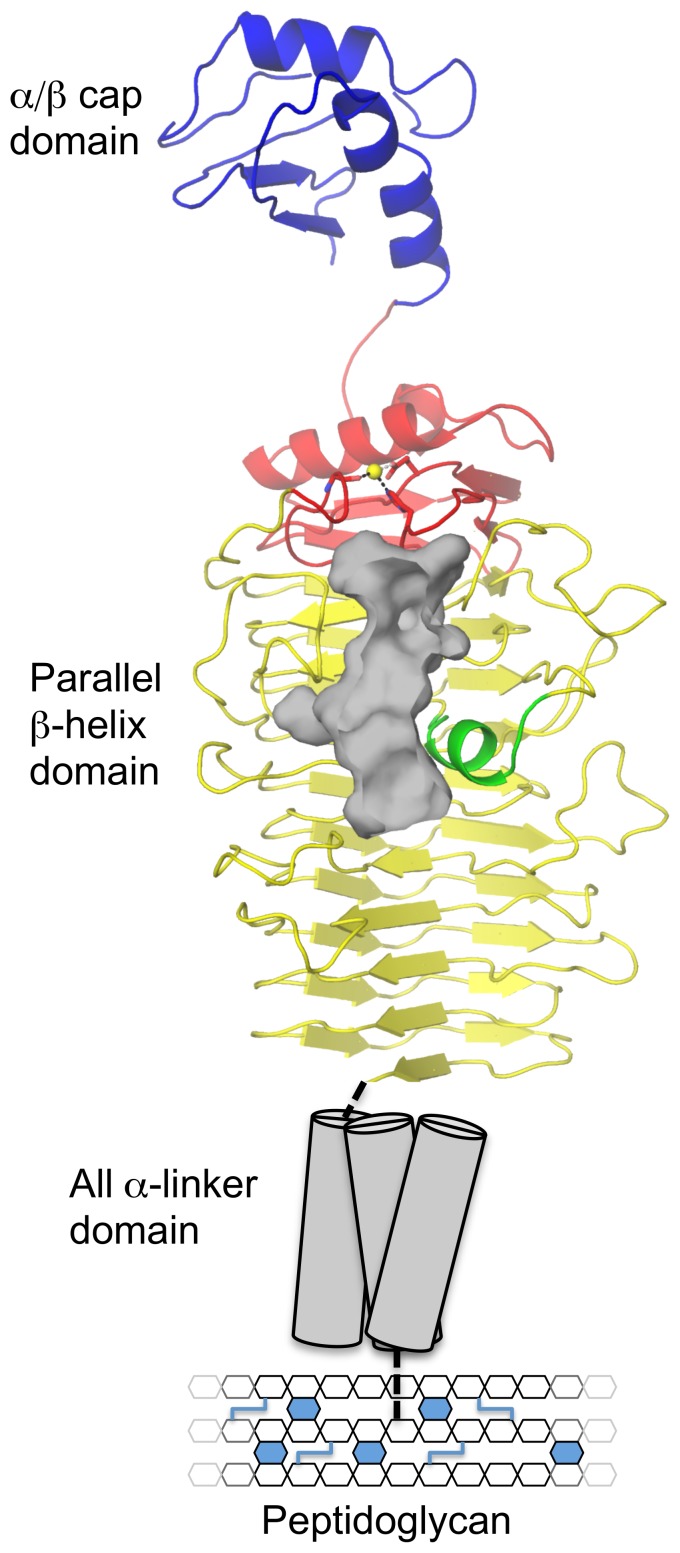
Overall model of PfbA. Utilizing a dissect-and-build approach of X-ray crystallography of the core domain (red, yellow and green) coupled with SAXs of the N-terminal (blue) and core domains, the overall structural features of the cell-wall attached pneumococcal adhesin PfbA reveal and overall elongated, bottle shaped structure. The cell-wall linking domain is schematically shown as tubes generally representing the predicted α-helical nature of this region. The putative carbohydrate recognition cleft is highlighted in grey.

Through its surface attachment and presentation PfbA is able to mediate the adherence of *S. pneumoniae* to host cells, likely through recognition of fibronectin [6]. Here we have provided insight into the structure of PfbA through the high-resolution X-ray crystal structure of the core parallel β-helix domain and the SAXS solution structure of this domain in tandem with the small N-terminal α/β-domain. Combining the available structural, bioinformatic, and biological data on PfbA results in a model of an elongated surface attached protein that has the capability of extending away from the bacterial cell-surface, perhaps optimizing recognition of its receptor (Figure 5). While the specific molecular details of the ligand for PfbA remain unknown, the parallel β-helix domain possesses features in its overall fold, a prominent surface groove, and even the arrangement of residues in the groove that are distinctly reminiscent of polysaccharide lyases. We were, however, unable to find such activity for the protein, suggesting the PfbAβ domain shares evolutionary ancestry with polysaccharide lyases but has lost this activity. Because of this structural relationship with carbohydrate-recognizing proteins and because the only common feature of the several known ligands of PfbA is that they are glycoproteins, we favor the hypothesis that PfbA is a novel family of carbohydrate-specific adhesins. Indeed, this would also be consistent with the known importance of glycan adhesion and processing in the virulence of *S. pneumoniae* [27].

## Materials and Methods

### Bioinformatics analysis

Bioinformatics analysis of the multi-modularity of PfbA from *S. pneumoniae* TIGR4 (SP_1833, NC_003028.3) was performed with Position Specific Iterative Blastp [28], SSM (pdbe.org/fold), Phyre [7], and I-TASSER [29]. Figures were generated using Pymol (pymol.org). For Consurf mapping of conserved residues onto the PfbAβ structure protein sequences with similarity to PfbA were identified with Blastp [28], representatives with significant similarity (e-value <10^−5^) were extracted via Blast Explorer (phylogeny.fr) [30], aligned with Mafft (mafft.cbrc.jp/alignment/server) [31], and an overall degree of consensus was mapped onto the crystal structure of PfbAβ (consurf.tau.ac.il) [32].

### Cloning, protein production and purification

The *pfbA* gene was synthesized with codon optimization for expression in *Escherichia coli* (Genscript). Using this source material as a template, the gene fragments encoding PfbAβ (amino acids 139-560) and a larger gene fragment encoding the N-terminal domain in addition to PfbAβ, called PfbAΔC (amino acids 52-560), were PCR amplified using specific primers to introduce a 5′ *Nhe*I and 3′ *Xho*I restriction sites (Table S1). The DNA encoding PfbAΔC and PfbAβ were cloned *via* the engineered restriction sites using standard molecular biology procedures into pET28a plasmid (Novagen), to generate plasmids pET28PfbAΔC and pET28PfbAβ, respectively. Expression vectors were transformed into *E. coli* BL21 Star (DE3) cells and recombinant proteins were produced using the autoinduction method [33] by shaking inoculated 1 L cultures supplemented with 50 µg/ml kanamycin for 36 hours at 37°C, reducing the temperature to 16°C, and continuing growth for an additional 48 hours. Selenomethionine substituted PfbAβ was similarly produced by growth in SelenoMethionine Expression Media (Molecular Dimensions) supplemented with 25 mM (NH_4_)_2_SO_4_, 50 mM KH_2_PO_4_, 50 mM Na_2_HPO_4_, 1 mM MgSO_4_, 0.5% w/v glycerol, 0.05% glucose, 0.2% α-lactose, pH 6.75, and 50 µg/ml kanamycin [33]. Cells were harvested by centrifugation and ruptured by chemical lysis procedure [34]. Clarified cell lysates were purified via nickel-affinity chromatography and fractions containing desired polypeptides were buffer exchanged by dialysis into 25 mM Tris-HCl (pH 8.0) prior to further purification by anion exchange chromatography. PfbAβ in 25 mM Tris-HCl (pH 8.0), 125 mM NaCl, and PfbAΔC in 50 mM Tris-HCl (pH 8.0), 500 mM NaCl were each concentrated using a stirred cell Amicon with a 10 kDa cutoff to 20 mg/ml. Protein concentrations were calculated by measuring absorbance at 280 nm using the molar extinction coefficients of 27850 and 29340 M^−1^cm^−1^, respectively for PfbAβ and PfbAΔC.

### Crystallization

All crystallization experiments were performed using the sitting drop vapor diffusion method at 18°C. Crystals of selenomethionine derivatized PfbAβ at a concentration of 20 mg/ml in 25 mM Tris-HCl (pH 8.0), 125 mM NaCl, were obtained by mixing 1.75 µl of protein with 1.75 µl reservoir solution consisting of 15% (w/v) 10 k polyethylene glycol, 0.1 M ammonium acetate (pH 7.0), and 10% (v/v) glycerol. Large (∼0.8×0.6×0.1 mm) plate crystals developed over a period of 3 days to a week. The single molecule form of PfbAβ at 20 mg/ml in 25 mM Tris-HCl (pH 8.0), 125 mM NaCl crystallized by mixing 1.0 µl of protein with 1.0 µl reservoir solution consisting of 12% (w/v) 10 k polyethylene glycol, 80 mM magnesium formate (C_2_H_2_O_4_Mg), and Bis-Tris-HCl (pH 5.5). In both cases, crystals were cryoprotected in crystallization solutions supplemented with 25% ethylene glycol and cryo-cooled directly in a N_2_ stream at −160°C prior to diffraction experiments.

### Data collection, Structure Solution and Refinement

Diffraction data from crystals of selenomethionine labeled PfbAβ was collected at the Stanford Synchrotron Radiation Lightsource beamline 9-2 and data from native crystals at the Canadian Lightsource beamline 08B1-1 (CMCF-BM). Both datasets were processed with iMosflm [35] and scaled with Scala [36]. The structure of PfbAβ was solved by SAD using data collected on crystals of selenomethionine derived PfbAβ at a wavelength optimized for the anomalous signal of selenium. The autoSHARP [37] workflow was used for selenium atom substructure determination, initial phasing, density modification and solvent flattening, followed by model building through iterative cycles of and density improvement and building with ARP/wARP [38]. The PfbAβ derivative structure was then improved with cycles of manual building with Coot [39] and refinement with Refmac [40]. This initial model was subsequently used as a starting model to solve the structure of the C222_1_ crystal form using Phaser MR [41] and this was complete by cycles of manual building and refinement. Five percent of the reflections were flagged as “free” to monitor refinement procedures. The final refined structure was validated using Molprobity [42]. Data collection and refinement statistics are presented in Table 1 and PfbAβ coordinates are deposited in the Protein Data Bank (pdb.org) under the accession code 3ZPP corresponding to the native structure.

### Small Angle X-ray Scattering

An additional purification step of size-exclusion chromatography in 50 mM Tris-HCl (pH 8.0), 500 mM NaCl was performed on PfbAΔC to remove aggregates, and samples were filtered through a 0.22 µm diameter membrane prior to Dynamic Light Scattering and SAXS analysis. SAXS data were collected at the SSRL beamline 4-2 using a Rayonix MX225-HE detector at 288 K with an X-ray wavelength of 1.5 Å. Sample exposure times were 1 s with 15 replicates, alternating with collection on a buffer standard, which served for background subtraction. The sample-to-detector distance was fixed at 1.7 m, leading to scattering vectors, Q, ranging from 0.01 to 0.4 Å^−1^. Solutions of PfbAΔC in 50 mM Tris-HCl (pH 8.0), 500 mM NaCl ranged in concentration from 1.0 to 2.5 mg/ml. A dilution series of bovine serum albumin was measured in addition to nuclease-free H_2_O as a reference and for scattering calibration and validation purposes. Background scattering was measured after each protein sample using the buffer solution and subsequently subtracted from the protein scattering patterns after proper normalization and correction for detector response.

SAXS data processing and determination of scattering-quality parameters R_g_, and D_max_ were performed as described previously [43]. The distance distribution function *P*(r) was calculated by the Fourier inversion of the scattering intensity *I*(q) using GNOM [23]. The *P*(r) function was also used to calculate the R_g_. The molecular mass of PfbAΔC was determined by using experimental data from a single SAXS curve on a relative scale and within a restricted q range as described by Fischer *et al.* [24]. *Ab initio* envelopes of PfbAΔC were generated with DAMMIF [25] using 10 independent runs without imposition of any shape constraints. The *ab initio* reconstructions were aligned, averaged, and filtered using the DAMAVER suite of programs [44].

The combined *ab initio* and rigid-body modeling program BUNCH was used to generate models from the scattering data using as rigid bodies the X-ray crystal structure of the PfbAβ domain (amino acids 139-559) and a model of the 84 amino acid N-terminal domain (amino acids 54-138), the latter of which was generated using the I-TASSER server [29], [45]. The solution scattering for the models, the fit to the experimental curve, and the goodness of fit was evaluated using CRYSOL [46].

### Activity assays

The potential polysaccharide lyase activity of PfbAΔC was assayed by spectrophotometry. Assays were performed with polysaccharide at 0.22% in a total volume of 500 µl containing appropriate buffer (100 mM of either Tris-HCl, NaH_2_PO4, or McIlvaine's buffer) and metal ions (5 mM CaCl_2_ or MgCl_2_). Reactions were initiated by the addition of protein to a final concentration of 20 µM. The formation of new unsaturated non-reducing ends was monitored by absorbance at 232 nm in a spectrophotometer at 37°C.

### Accession Codes

Coordinates and structure factors have been deposited in the protein data bank with the following accession codes: 3ZPP.

## Supporting Information

Figure S1 **Ribbon (A) and cartoon (B) structural alignments of PfbA and homologues.** The structure of the PfbAβ domain (yellow) shares significant structural identity with the *endo*-*N*-acetylglucosaminidase tailspike protein from the *E. coli* bacteriophage HK620 (red: r.m.s.d. = 2.08 Å with PDBID 2VJJ over 308 residues) [11], the *Pedobacter heparinus* Chondroitinase B (grey: r.m.s.d. = 2.18 Å with PDBID 1OFL over 278 residues) [12], the Bacillus sp. snu-7 inulin fructotransferase (blue: r.m.s.d. = 2.18 Å with PDBID 2INV over 245 residues) [13] and the pectate lyase Pel9A from *Erwinia chrysanthemi* (green: r.m.s.d. = 2.37 Å with PDBID 1RU4 over 254 residues) [14].
(PNG)

Table S1 **Oligonucleotide primers used for amplification and cloning.**
(DOC)
