# Supplementary figures and images for: Structure of the Streptococcus pneumoniae Surface Protein and Adhesin PfbA
Source: PLoS One. 2013 Jul 22;8(7):e67190. doi: 10.1371/journal.pone.0067190 (PMC3718772; doi:10.1371/journal.pone.0067190)

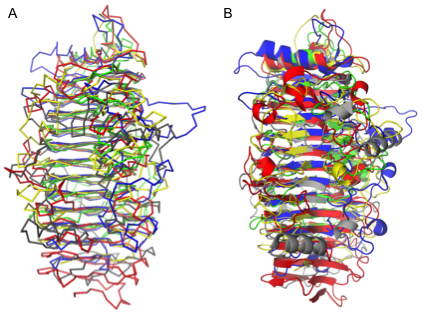

Supplement: Figure S1 — Ribbon (A) and cartoon (B) structural alignments of PfbA and homologues. The structure of the PfbAβ domain (yellow) shares significant structural identity with the endo-N-acetylglucosaminidase tailspike protein from the E. coli bacteriophage HK620 (red: r.m.s.d. = 2.08 Å with PDBID 2VJJ over 308 residues) [11], the Pedobacter heparinus Chondroitinase B (grey: r.m.s.d. = 2.18 Å with PDBID 1OFL over 278 residues) [12], the Bacillus sp. snu-7 inulin fructotransferase (blue: r.m.s.d. = 2.18 Å with PDBID 2INV over 245 residues) [13] and the pectate lyase Pel9A from Erwinia chrysanthemi (green: r.m.s.d. = 2.37 Å with PDBID 1RU4 over 254 residues) [14]. (PNG) [file pone.0067190.s001.png]
