# Supplementary material for: Structure of the Streptococcus pneumoniae Surface Protein and Adhesin PfbA
Source: PLoS One. 2013 Jul 22;8(7):e67190. doi: 10.1371/journal.pone.0067190 (PMC3718772; doi:10.1371/journal.pone.0067190)
Supplement: Table S1 — Oligonucleotide primers used for amplification and cloning. (DOC) [file pone.0067190.s002.doc]

Supplemental Table 1: Oligonucleotide primers used for amplification and cloning.

| **Oligonucleotide** | **Sequence** | **Used to Amplify and Clone** |
| --- | --- | --- |
| PfbA-Ser52For | CAT ATG GCT AGC TCA AAC GCC ATC ACG AAT GAT C | PfbAΔC |
| PfbA-Thr139For | CAT ATG GCT AGC ACC GCG CTG AGC GTT AAA G | PfbAβ |
| PfbA-Ser560Rev | GTG GTG CTC GAG TTA GCT TTT GAT GAA CGA AAT G | PfbAβ / PfbAΔC |
|  |  |  |
